# Art, fisheries and ethnobiology

**DOI:** 10.1186/1746-4269-11-17

**Published:** 2015-02-23

**Authors:** Alpina Begossi, Rodrigo Caires

**Affiliations:** UNICAMP: CAPESCA/NEPA, Rua Albert Einstein 291, Campinas, SP 13083-852 Brazil; FIFO/ECOMAR/UNISANTA, Santos, SP Brazil; MZUSP, São Paulo, SP Brazil

## Abstract

**Background:**

Nature is perceived in a variety of forms, and the perception of nature can also be expressed in different ways. Local art may represent the perception of nature by humans. It can embody perception, imagination and wisdom. Local art, in particular, reflects how people interact with nature. For example, when studying the representation of fish by different cultures, it is possible to access information on the fish species found in the environment, on its relative importance, and on historical events, among others. In this context, art can be used to obtain information on historical events, species abundance, ecology, and behaviour, for example. It can also serve to compare baselines by examining temporal and spatial scales. This study aims to analyse art and nature from a human ecological perspective: art can understood as an indicator of fish abundance or salience.

Art has a variety of dimensions and perspectives. Art can also be associated with conservation ecology, being useful to reinterpret ecological baselines. A variety of paintings on fish, as well as paintings from local art, are explored in this study. They are analyzed as representing important fish, spatially and historically.

**Methods:**

A survey regarding the fish found in different paintings was conducted using art books and museum books. Pictures were taken by visiting museums, particularly for local or traditional art (Australia and Cape Town).

**Results:**

The fish illustrated here seem to be commonly important in terms of salience. For example, *Coryphaena* spp. is abundant in Greece, Nile tilapia in Egypt, *Gadus morhua* in the Netherlands, as well as barracuda in Australia; salience is also applied to useful, noticeable or beautiful organisms, such as *Carassius auratus* (China). Another aspect of salience, the diversity of a group, is also represented by the panel where *Uraspis uraspis* appears to be depicted.

**Conclusions:**

Regarding the evaluation of baselines, we should consider that art may represent abundant fish in certain historic periods and geographic regions. Art could be an important temporal and geographical indicator to discover preterit information on the abundance of fish and compare it to present abundance.

## Introduction

Art has a variety of dimensions and perspectives. For example, it can be approached as *connoisseurship*, such as in the aspect of when and where it was made, as well as by whom; as *historic style*, such as by interpreting the style in the historic context; as *social history*, by analysing the social conditions by which different styles and forms appear; as a *symbol*, such as interpreting it in its religious, ritual or symbolic contexts; as a *psychological aspect*, by assessing perception and psychological theories associated with art; as *taste*, representing its collection perspective; and as a *technique*, such as painting techniques and tools, among others [1]. Frothingham [2] when exploring some conceptual issues of art, observed that in every period of civilization, the universal ideas that ruled the period were externalised by multiple forms in the different interrelated arts from philosophy to painting. This concept may parallel the concept of *Zeitgeist* (see also Hegel) [3]. The author, who lived during the XIX century, comments upon the mission of art as being historic, interpretive, and creative.

Throughout history, different definitions of art and nature can be observed. As noted by Morris [4] the philosopher believes art is the true substance; the artist feels, loves and is moved by art. As the author stressed, art does not simply imitate nature but idealises it. Plato’s ^a^ definitions consider art as inferior to nature. Aristotle argues that nature is form and matter, the organisation of human art is based on form over matter in nature, and art imitates nature (nature and art are considered as parallel creative processes: art imitates nature and vice-versa) [5]. These ideas, as observed by Close [5], reappear in the Renaissance period. In the Enlightenment, art and science were connected (helping to narrow the meaning of art) [6]. Frothingham [2] discloses art as imitating nature by understanding it as a product of man’s creative intelligence. The period of Frothingham’s work was certainly a fertile period where art was separated from the idea of imitating nature. Eventhough a natural tendency for a general observer is to be tempted to view 'reality’ represented into paintings. As observed by Carroll [7] when looking at human nature, art appeared independently at different sites and times.

Recently, an aspect of art associated with conservation ecology has come to light. The importance of shifting baselines and ancient art work were suggested as tools for acquiring information on fish [8]. The notable work of Guidetti and Micheli [9] showed that the analysis of ancient art can be useful to reinterpret ecological baselines. In this regard, as observed by these authors, some fish species such as grouper may have had larger body sizes in the past. Guidetti and Micheli [9] analysed more than 73 ancient Roman mosaics, with 23 from the 1^st^-5^th^ centuries B.C. As illustrated, Micheli [10] observed: “*When we considered a species recovered*, *they may still in fact be altered relative to their original baseline*” (*Stanford News*, *September 7*, *2011*). Therefore, ancient history can be helpful in reinterpreting baselines of fish sizes and schooling.

Following the idea of linking art, fisheries and the environment, we focus on baselines. Baselines are a reference point by which stocks are compared, and then the increase or decrease in the baseline is evaluated. Now, considering fisheries, fish and conservation, we recall the classic study of shifting baselines by Pauly [11]. The theory states that depending on the time period when fish stock data are collected, the collected data may actually represent a referenced baseline. Therefore, our current data on fish stocks are compared to baselines of these stocks that refer to the first set of data or the year when the data were collected. Bender *et al*. [12] applied this concept to local ecological knowledge (LEK). In this context, local or traditional communities have an idea of the stocks, as well as their past (information from elders in the community) and current status. Therefore, if this information can be accessed through surveys on local historical accounts, then it is also possible to access earlier baselines on specific stocks. They studied such baselines based on the LEK of 9 species, 3 of which were groupers (*Epinephelus*) off the coast of Bahia, Brazil (locality of Porto Seguro). The shifting baselines concerning these species were then observed by comparing information obtained among fishermen from 40 years ago to the present.

Finally, we highlight the study of Guidetti and Micheli [9], in which earlier baselines were most likely accessed through antique art, especially mosaics. We define the objectives of this study as an exploratory and preliminary survey accounting for the following:

- information on fish observed in art at different ages and locations. We will initially concentrate on paintings/drawings.
- information on fish from local drawings and paintings within different, current traditional cultures.

## Results

### Art and Fisheries: exploration throughout ages and locations

**Figure 1:**
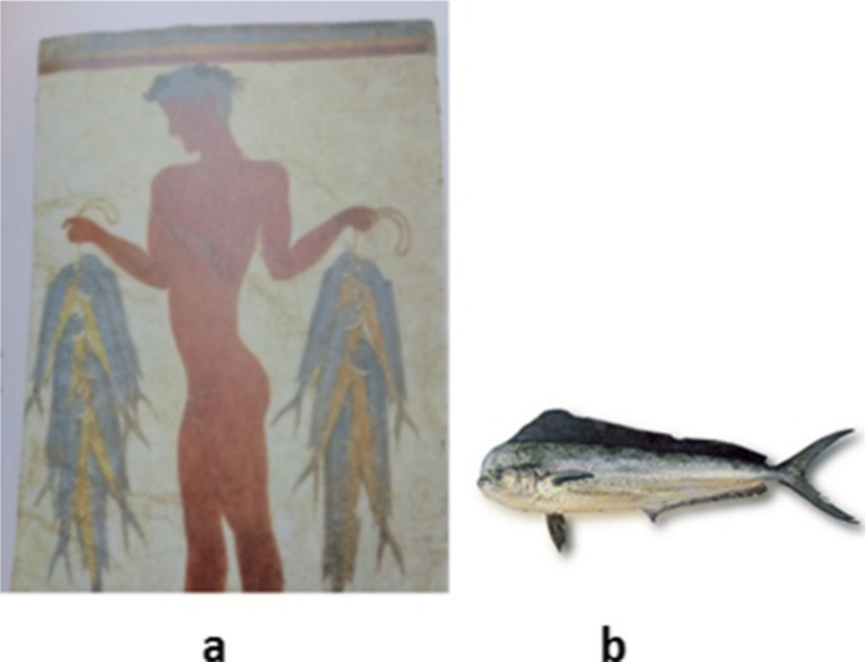
**Image from Santorini and of** ***Coryphaena.*** **(a)** From Santorini, 1500 BC [1]. *Coryphaena hippurus* or *C. equiselis*? **(b)** *Coryphaena hippurus* (http://www.fao.org/fishery/species/3130/en). Image in [1] and in [8].

**Figure 2:**
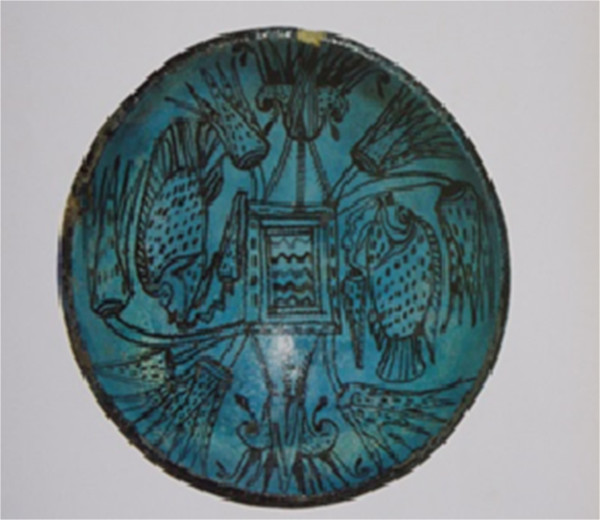
**Egyptian plate,** **XIX Dynast,** **XIII century B.C.** (In Ragghianti [14], p 141; Cairo Museum).

**Figure 3:**
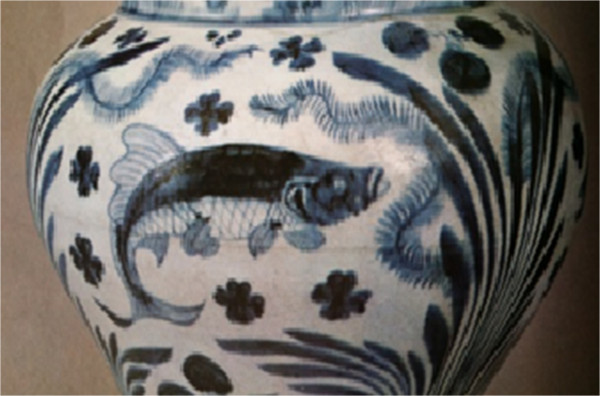
**Detail of a vase of Yuan Dynasty,** **China** **(** **XIII** **-** **XIV centuries** **).** In Ragghianti [22].

**Figure 4:**
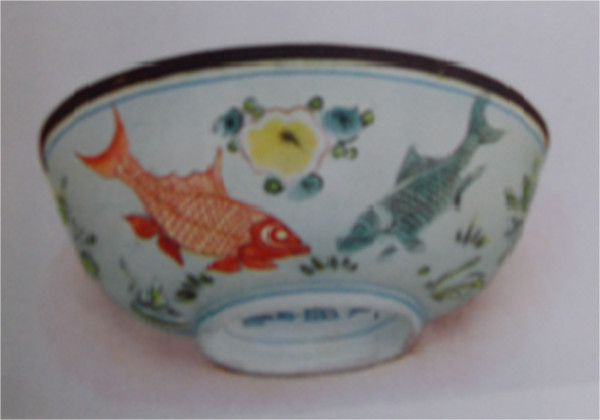
**Porcelain,** **Ming Dynasty,** **Kia**-**tsing period,** **1522** **–** **1566** **(** **XVI century** **).** In Hobson [19].

**Figure 5:**
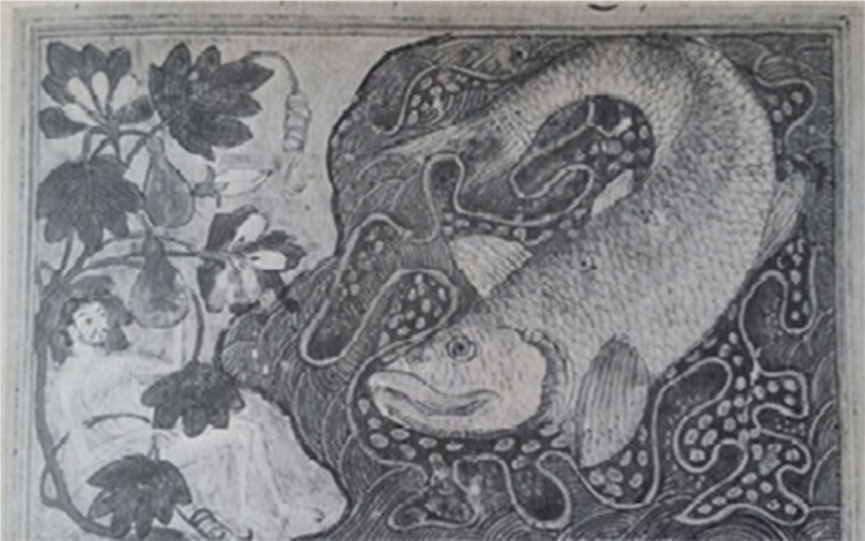
**Illustration from the Edinburgh Rashid** **-** **al Din,** **'** **Universal History** **’,** **1306,** **Persia [** [20] **]** **.**

**Figure 6:**
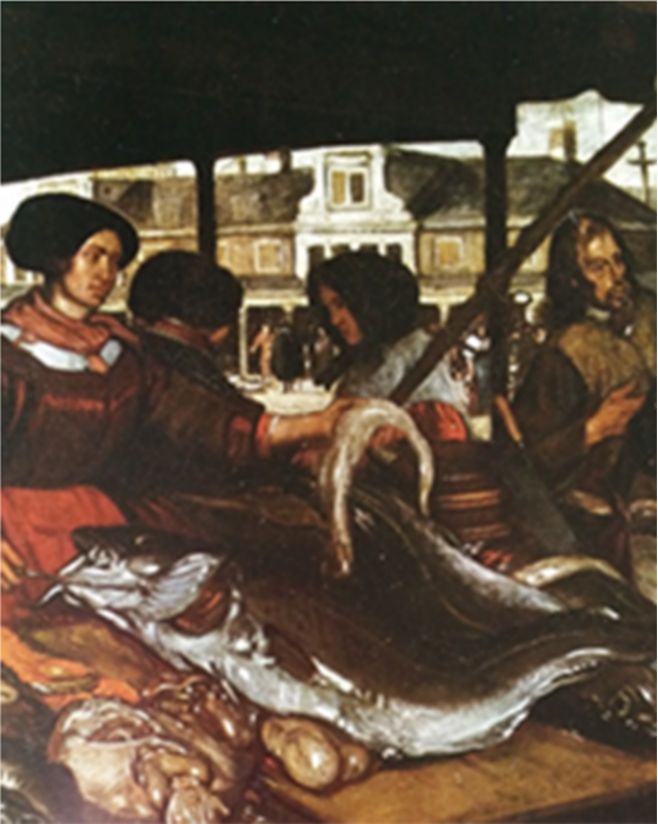
**Detail of Emanuel De Witte**’**s The Fish market.** Oil painting, approximately 1672 [24].

**Figure 7:**
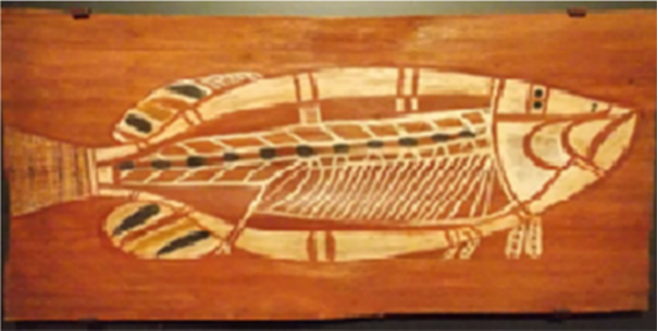
**Barramundi** **(** ***Lates calcarifer*** **)** **in illustration** **(** **bark painting** **)** **at the Australian Museum,** **Sydney,** **Australia,** **January 2013.** “*Artist unknown*; *Croker Island*, *West Arnhem*, *Northern Territory*; *Acquired 1965*; *Barramundi are an important food source for the communities of Croker Island*; *during March and April*, *Barramundi swim over flood plains and are caught with large specially designed traps or spears*”.

**Figure 8:**
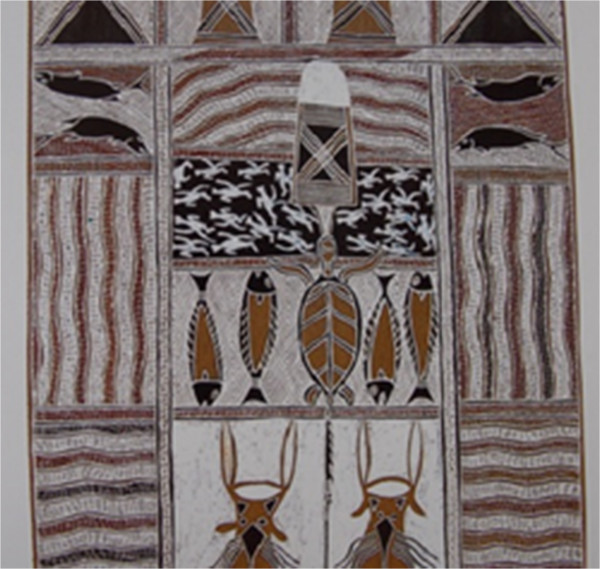
**Detail of bark painting by Baluka Maymuru,** **a painting of Mayawundji in Djarrakpi,** **Blue Mud Bay,** **Maritime Museum,** **Sydney,** **Australia,** **January 2013.**

**Figure 9:**
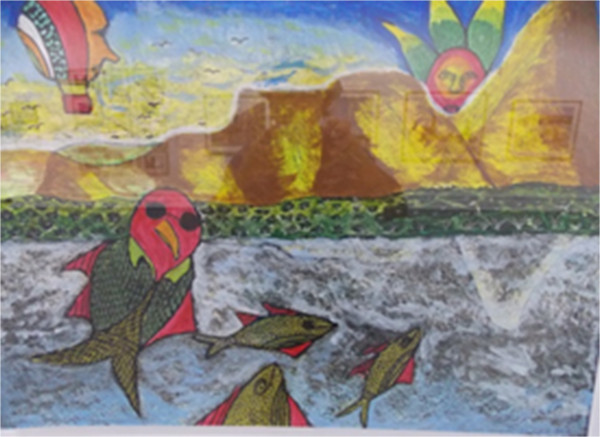
**Detail of** **'** **Reflection** **’** **by Thabila Dubula** **(** **2012** **),** **South Africa Museum,** **Cape Town,** **January 2013.**

**Figure 10:**
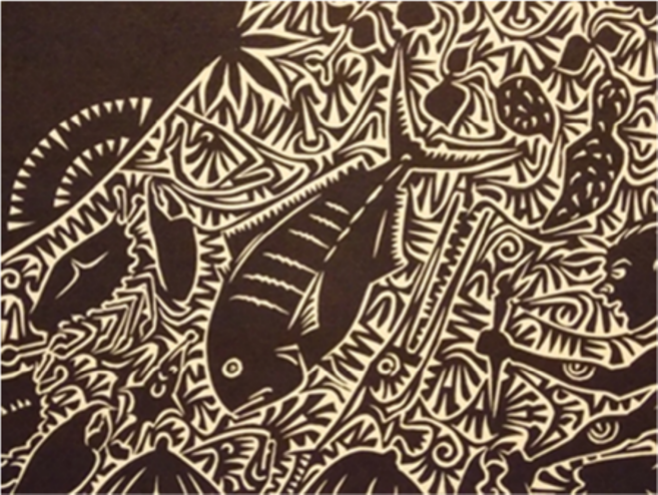
**Detail by Alick Tipoti,** **1975,** **Torres Strait;** ***Gubal Aimai Mabaigal*** **(** ***wind makers season*** **);** ***linocut printed in black ink from one.*** In Art Gallery Cairns, Australia. January 2013. Exposition relative humidity: a Cairns Regional Gallery Exibition.

We will explore a variety of paintings on fish, as well as some local art, as a preliminary exploratory analysis. We include examples from different parts of the world. Figure 1, Figure 2, Figure 3, Figure 4, Figure 5, Figure 6, Figure 7, Figure 8, Figures 9 and 10 also reveal other examples throughout history of fish paintings that may be useful to re-interpret baselines. Beginning with a painting from Santorini (Figure 1), Greece, we observe a fisherman with many *Coryphaena* specimens caught. As dolphinfishes are gregarious species, being common in the Mediterranean area, this image probably represents the result of a catch.

Although the bright yellow coloration on the belly indicates that the artist represented *C. hippurus*, which is fairly common in Greece and called Dakaunomoutas, Kynigòs, or Kynygós by native fishermen (common dolphinfish in English)(http://www.fao.org/fishery/species/3130/en), it is not possible to confirm this identification. *Coryphaena equiselis*, the only other dolphinfish species, usually has a paler yellow hue on the flank, but the main diagnostic characters for each species come from ray counts, pelvic fin colour and anal fin shape [13], which are not visible in the above illustration. Eventually, *Coryphaena* is common in Greece, what makes that painting possibly representative in a temporal and geographical scale. This Figure has been commented [8] and suggested that this species was probably more abundant from now.

Figure 2 refers to an Egyptian ware plate of the XIX Dynasty, XIII B.C. The period is Ramsés I or II or Seti I or II, among others of the XIX Dynasty. The blue ware was composed of a sandy paste covered by a siliceous varnish, and aquatic themes were common. The palms were a theme imported from Syria [14]. The fish represented in this figure is Nile tilapia, *Oreochromis niloticus*. Nile tilapia is the most important cichlid species in inland Egyptian fisheries; since ancient times, it has been an important food source [15].

Figure 3 is a Chinese vase, porcelain (Yuan Dynasty). This technique started in the Han period (206 BC-221 AD), and it was an important material of the 'Six Dynasties’ (221–586 AD). It was carried into the Yuan period, which is the period of the vase, and ornamented with fish and algae. In this period, techniques related to blue ceramics were imported from Persia [16]. The fish illustrated in this figure is most likely the native goldfish *Carassius auratus*. It is a freshwater fish, distributed widely in and around the Eurasian continent, including Taiwan and the Japanese islands. Goldfish are very well known in ancient China. They were first described in ancient Chinese writings 2750 years ago (over 100 varieties exist) [17]. During the Jin Dynasty (265–420 AD), the mutated colours were observed, and ornamental water gardens were shown later during the artistically inclined Tang Dynasty (618–907 AD). During social gatherings, some of the finer specimens were temporarily show cased in smaller containers – the world’s first fish tanks – to show off to guests [18].

Figure 4 shows a Chinese porcelain of the Ming Dynasty from the Kia-tsing period (1522–1566) [19]. The two fish illustrated in this figure are also different varieties of the goldfish *Carassius auratus*.

An illustration from the Edinburgh Rashid-al Din, 'Universal History’, 1306, Persia, represents Jonah from the Old Testament (Figure 5). The Rashid-al Din (*Jami al*-*Tanarikh*) has four volumes with this drawing as one of the hallmarks of the Tabriz School (Ilkhanid dynasty) [20]. During the Ilkhanid period, a Mongolian period in Persia, arts were especially developed and included manuscript illustration. This is a special illustration because it embodies a tricky interpretation: it is the depiction of Jonah and the whale from 1306. What makes this illustration tricky is that we see a large fish and not a whale. However, there is a debate over the large fish ('*the big fish*’) [21]: within the bible text, it might have been a whale, a shark, a sea monster or a large fish; however, it most likely represents a large fish (*dag gadol* in Hebrew) [21]. From the illustration, we could not confidently identify the fish species that is represented. The drawing resembles the Chinese porcelain objects included above in the head shape and single dorsal fin and large scales, differing only in some details of the dorsal and anal fin shape, size and origin; *Carassius auratus* is not native to Iran and was introduced in this country after the XIV century [22]. Other fish taxa that are somewhat similar to the species depicted in the illustration are some members of the order Clupeiformes (sardines and allies [23]), although the abdominal scutes that are characteristic of this group cannot be observed in the image.

It is not clear whether the artist actually intended to represent a specific fish species or if this drawing is an allegory (or archetype whatsoever) of a giant fish in the artist’s mind.

The detail observed in the painting by Emanuel De Witte, *The Fish market*, which is an oil painting from approximately 1672 [24], is a fish (Figure 6). Abundance is also a feature of the fish represented in this painting, as observed in some other previous illustrations. The fish depicted in this image is certainly *Gadus morhua* (cod) for the following reasons: 1) certain diagnostic features of this genus can be observed in the painting, such as the long chin barbel and two separate anal fins; and 2) *G. morhua* is the most frequent of the three known species of this genus and the only one that is encountered on the coast of the Netherlands, which is the country where De Witte lived (see also Cohen *et al*. [25]).

### Art and Fisheries: local art or art of current traditional people

The pictures included below are from local art, such as examples of aboriginal art from Australia, as well as a drawing from a Museum in Cape Town, Africa.

The art shown in Figure 7 is from Australia, most likely represents the barramundi, *Lates calcarifer*, which has been commonly painted in aboriginal art. It is the most important Australian commercial fish [26]. Therefore, the aspect of abundance and local art is demonstrated through the common painting of the barramundi in aboriginal art.

In Figure 8 there is a bark painting photographed at the Maritime Museum of Sydney. It was painted by Baluka Maymuru, a leader of the Mangalili country, located in Djarrakpi, Gulf of Carpentaria, and represents the flow of sacred waters from the Maywundji into the salt water of Milniyawuy from the bottom-up; in this panel, the artist connects the clan territories of land/salt water/deep waters of the sea along with the elements within [27]. It is difficult to assign the fishes depicted in the image to any taxonomical element, although it is feasible to suggest that the species may be mackerel (family Scombridae) because of their elongate shape and dorsal and anal fins that elude the pinnulae found in members of this family. Interestingly, Spanish mackerel is a very important catch, accounting for 40% of the total catch, which included 75 species in the sampling period from 1984–1986 at Yorke Island, Torres Strait [28]. In particular, as reported by these authors, Spanish mackerel and green turtles accounted for 65% of the landings. Further, the local drawings represent abundant species in these examples.Figure 9 is a drawing from South Africa Museum at Cape Town, and identification for this drawing is not possible. This is an example of a drawing in which more information would be needed either from the artist or from locals for taxonomic identification.

Figure 10 is a painting from the Torres Strait from an Art Gallery in Cairns, Australia. The fish presented in the illustration is possibly a carangid. Light vertical stripes, similar to those observed in the image, are present in members of the genus *Uraspis* (cottonmouth trevally).

*Uraspis uraspis* (white-tongued jack) has been reported in the Northern Territory coast [29].

In a study in the Gulf of Carpentaria, *Uraspis uraspis* accounted for 18% of the catch (frequency of occurrence in experimental trawls) [30]. The Carangidae, the family of *U. uraspis*, is very diverse and abundant in Australia [31].Therefore, this fish, even if not highly abundant, is representative of a high diverse fish family in this region (Carangidae). We should also consider richness, an aspect of diversity expressed by the number of species, as a component of perceptual and ecological salience.

## Conclusions

The fish illustrated here seem to be commonly important in terms of salience. They are images of fish, but those images represent important, or more precisely, abundant fish within their origin locations. For example, *Coryphaena* spp. is abundant in Greece, Nile tilapia in Egypt, *Gadus morhua* in the Netherlands, as well as barracuda in Australia; salience is also applied to useful, noticeable or beautiful organisms. This criterion applies for the Chinese paintings, where *Carassius auratus* is depicted. Another aspect of salience, the diversity of a group, is also represented by the panel where *Uraspis uraspis* appears to be depicted.

One important aspect to note is that our method was independent in terms of choosing a fish or a region. We consulted available books of art, mostly following museum books and some art books, which could be geographically related such as 'Art Chinois’ [19] or Islamic Art [20]. Thus, the images selected here show that, in a preliminary analysis, most of the fish shown and identified in the figures are abundant fish or represent a group where there are other similar fish with high richness (high diversity) or any other salience, such as in the Chinese case of ornamental fish.

These results are a preliminary indication that art could represent the perception of salient features of nature. Salience is an important aspect of studies of perception and linguistics, and the concept has been an important approach for the understanding of ethnotaxonomy, or the taxonomy of local or traditional populations.

Thus, the salience of both ecological (abundant fish) and cultural (ornamental fish) aspects appears to be associated with the fish images painters represent, as shown by the examples in this study. Salience refers to noticeable, conspicuous, or culturally important attributes of some species over others. Hunn [32] observed that abundant and widely distributed organisms are more likely to be noticed than those rare, narrowly distributed species. Additionally, size affects perceptual salience, such as readily visible organisms. Other organisms also form search images of food that might be appreciated or avoided diet items. For example, birds can form search images on aposematic insects, such as beetles, and those prey species are avoided due to the bad taste from terpenes or other secondary substances found in their host plants [33, 34]. Thus, search images can make nature selective for perception. This perception might be a stimulus for representing these animals in art. Art, within a context of time and space, can be helpful in representing baselines in nature (in this case, related to fish).

Brown [35] analysed salience in detailing its importance in the perception and categorisation of organisms in nature. Atran [36] observed that the salience of life forms has repercussions on the ability to use this appreciation in the life context of local nature: our perception of groupings in nature, for example, might be facilitated by our own restrictive possibilities of perception (focal colours, bodily objects, temporal relations, among others). Mental images can be formed based on perception and intellectual processes (*Gestalten*[37]). As Hunn [32] described, perceptual or cultural salience includes aspects of an organism that when under external stimuli, determine the likelihood of what will be perceived and thus categorised. Thus, abundance, size, colourfulness, beauty, ugliness, usefulness for consumption, commerce, medicine or even poisonous animals might fall in this category. In ethnotaxonomy, debates have been carried out on the importance of salience in perceiving and classifying nature. In the context of this study, we bring the hypothesis that art also represents, at least in part, salient organisms. Berlin [38] observed that cultural importance was a feature of salient organisms, and Brown [35] observed the relationship of discontinuities in nature marked by features of attribute clustering/gestalt properties/attributes of discontinuities/salience dimension. Features that facilitate the easy recognition of important organisms, ecologically and culturally speaking, should facilitate the search images and thus be salient.

However, what impact or consequence could art have on the sustainability of fishes? Regarding the evaluation of baselines, we should consider that art may represent abundant fish in certain historic periods and geographic regions. Thus, art could be an important temporal and geographical indicator to discover preterit information on the abundance of fish and compare it to present abundance.

## Methods

A survey regarding the fish found in different paintings was conducted using art books and museum books (see References). Pictures were taken by visiting museums, particularly for local or traditional art (Australia and Cape Town). The sources are cited in Figures 1, Figure 2, Figure 3, Figure 4, Figure 5, Figure 6, Figure 7, Figure 8, Figure 9 and 10.

The survey was performed per museum book (and other available books of art) and not per fish.

### Endnotes

^a^Plato’s in the Republic classified three universal types of creators, illustrating the falsity of the mimetic representation of art (God – the ideal; carpenter – he models his artefact; and the painter – not original, as he makes copies) [5].
